# Design, Implementation, and Preliminary Evaluation of an Undergraduate Nursing Informatics Literacy Course Based on the ADDIE Model: A Single-Arm Mixed-Methods Study

**DOI:** 10.3390/nursrep16050151

**Published:** 2026-04-28

**Authors:** Huina Zou, Linjing Wu, Kaixin Li, Polun Chang, Yuan Chen

**Affiliations:** 1 Xiamen Cardiovascular Hospital, Xiamen University, Xiamen 361000, China; zouhuina@xmheart.com (H.Z.);; 2 Institute of Biomedical Informatics, Taiwan Yang-Ming Chiao-Tung University, Taipei 112304, China

**Keywords:** informatics competency, nursing informatics, nursing students, ADDIE, undergraduate

## Abstract

**Background/Objectives**: Nursing informatics competency is critical for nursing students entering clinical practice in the digital era. Current undergraduate nursing informatics courses prioritize theoretical instruction but lack sufficient integration with clinical applications, which restricts the depth of content delivery. This study aimed to design, implement, and conduct a preliminary evaluation of an undergraduate nursing informatics literacy course. **Methods**: This was a single-arm mixed-methods study. We implemented five sequential steps aligned with the Analysis, Design, Development, Implementation, and Evaluation (ADDIE) model: (1) needs assessment, (2) design of a systematic, progressive course spanning theoretical foundation, technical skills, and clinical application; (3) development of teaching materials and environment; (4) implementation through nine weekly 90 min sessions incorporating teach–practice–feedback; and (5) evaluation via a customized questionnaire and nursing informatics system project reports. Quantitative data were analyzed using the mean, standard deviation, and paired *t* tests; qualitative data were collected through post-course semi-structured interviews. **Results**: A total of 120 participants were enrolled from a provincial public medical school, of which 119 (99.2%) completed the course. Statistically significant pre–post improvements were observed in participants’ nursing informatics competencies, informatics literacy self-efficacy, and innovative behavior after course completion (*p* < 0.001). Participants completed 12 group projects, which received two C grades, nine B grades, and one A grade. Overall course satisfaction averaged 89.03 ± 14.62. Qualitative interviews yielded three themes: (a) cognitive reconstruction and improvement in the ability to apply nursing informatics competencies, (b) dilemmas and breakthroughs in the integration of technology and knowledge in nursing informatics design, and (c) optimization direction of course content, design, and evaluation. **Conclusions**: An ADDIE-based nursing informatics literacy course may be associated with improvements in nursing informatics competencies, informatics literacy self-efficacy, and innovative behavior among nursing undergraduates. This study provides preliminary evidence supporting the feasibility and potential educational value of such a curriculum.

## 1. Introduction

As technology becomes increasingly integrated into healthcare systems, nurses need a strong informatics foundation to work effectively in digital care environments [1]. Therefore, nursing informatics competency is essential for students preparing to enter modern clinical practice [2]. Although there is no internationally harmonized definition of nursing informatics competencies, a comprehensive framework that clearly defines knowledge, skills, and attitudes has been established [3]. Nursing informatics competency enables nurses to collect, analyze, and interpret data. This supports clinical decision-making, improves patient outcomes, and fosters interdisciplinary communication via electronic health records [4,5]. Several countries with advanced nursing informatics have integrated nursing competency training into undergraduate and higher-level nursing courses [6,7]. Simultaneously, many organizations have issued guidelines for nursing informatics education. For example, Technology Informatics Guiding Education Reform (TIGER) has developed a set of competencies to guide professional development in nursing informatics [8]. However, surveys have indicated that undergraduate nursing students’ informatics competency remains low to moderate [9,10].

### Background

In recent years, nursing informatics education research has expanded to include information literacy, clinical information system application, innovative teaching methodologies, and curriculum reform [6,11,12,13,14]. Sockolow and Bowles [15] trained students studying for a Master of Science in nursing using instructional strategies for teaching systems-development-lifecycle project management and guided them to complete an informatics system-related project report. Kim [16] integrated web development skills into a master’s program in nursing informatics and achieved high satisfaction with the program. These studies did not directly measure nursing informatics competency, so their real-world effectiveness is unclear. Most programs targeted graduate students, creating a need for effective undergraduate strategies [17]. Li et al. [18] developed a nursing informatics course with project-based learning for undergraduates (24 students), which improved students’ informatics acceptance attitudes and skills, but lacked clinical relevance and practical orientation. In addition, Shen et al. introduced design-based learning (DBL) into undergraduate nursing informatics education and assessed its efficacy in 622 nursing undergraduates. Real clinical problems and repeated practice were used to guide learning. The study showed that DBL could improve students’ information literacy, interdisciplinary thinking, and teamwork skills. However, the study depended on long-term repeated improvement and clinical practice support through university–hospital collaboration. The wider use of this course still needs further testing. A scoping review of nursing informatics and undergraduate nursing curricula reported that barriers to effective engagement with and proficiency in nursing informatics persisted, including a lack of understanding of nursing informatics, limited infrastructure and resources, inadequate digital literacy among students and faculty, and the evolving nature of nursing informatics [13]. These findings suggested a gap between current educational courses and the competencies required in clinical practice [19,20]. Thus, future research should focus on optimizing course content and teaching methodologies, and increasing hospital-based practical exposure (e.g., clinical visits) in nursing informatics education.

Bandura’s Social Cognitive Theory identifies self-efficacy as the motivational basis for skill development and behavior [21]. In this study, informatics literacy self-efficacy was the main motivational driver. This construct describes an individual’s confidence in acquiring, evaluating, and applying information. It may affect learners’ willingness to actively explore information, and is also closely related to knowledge transfer and informatics ability [22,23]. Moreover, some studies have found that informatics competence is positively correlated with innovative behavior [24,25]. Innovative behavior refers to an individual’s capability to generate original and potentially useful ideas, including the process of applying those new ideas in practice [26]. It is a key driver for translating new information, knowledge, and technology into concrete nursing practices and optimizing care processes. Previous studies have suggested possible associations among self-efficacy, informatics competencies, and innovative behavior [27]. These constructs were therefore used as a conceptual framework to guide outcome selection in the present study. However, the relationships among them were not empirically tested in this study. Conceptually, self-efficacy may motivate learning engagement, competencies may support practical application, and innovative behavior may reflect higher-order use of acquired knowledge and skills. Based on this theoretical relationship and the needs of clinical digital nursing, this study selected these three domains as the core outcome indicators to evaluate the course’s effect. However, intervention studies and outcome evaluations on these three indicators in undergraduate nursing education remain limited. Owing to limited learning opportunities, undergraduate nursing students generally perform worse than postgraduate students in these indicators [22,23,24,28]. This gap requires a structured intervention.

Therefore, nursing educators should develop a more systematic, skills-oriented nursing informatics course tailored to undergraduates’ needs, thereby narrowing the gap between theory and practice [25]. Moreover, the course should focus on the integrated intervention of nursing informatics competencies, informatics literacy self-efficacy, and innovative behavior of nursing undergraduates. The ADDIE model is primarily a training framework grounded in learners’ needs [29]. It consists of five stages, namely Analysis, Design, Development, Implementation, and Evaluation. Researchers can use it to analyze learners’ needs, learning content, and characteristics; select the most suitable training methods for learners; and make the course system more systematic and effective, thereby further mobilizing learners’ enthusiasm and initiative. Accordingly, this study aimed to design, implement, and preliminarily evaluate an ADDIE model-based nursing informatics literacy course for undergraduate nursing students. The specific objectives were: (1) to develop a systematic curriculum integrating theoretical foundations, technical skills, and clinical applications; (2) to preliminarily evaluate the effects on students’ informatics competencies, informatics literacy self-efficacy, and innovative behavior; and (3) to explore student learning experiences to inform course optimization.

## 2. Method

### 2.1. Design and Setting

**Figure 1:**
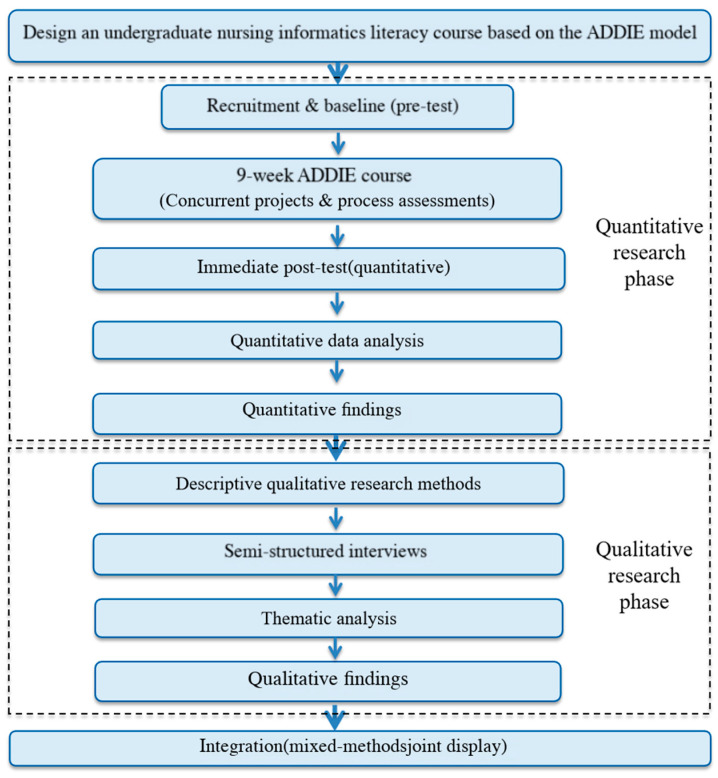
Procedural diagram of the explanatory sequential mixed-methods design.

This was a single-arm, pre-test–post-test mixed-methods study. We preliminarily evaluated short-term outcomes of an ADDIE-based nursing informatics literacy course among first-year undergraduate nursing students at a public medical university. The study was conducted from March to June 2025. We adopted an explanatory sequential mixed-methods design (Figure 1). We first used quantitative pre–post measures to objectively assess short-term changes in informatics competencies, self-efficacy, and innovative behavior. Next, we purposively recruited a diverse subset of participants for semi-structured interviews. These interviews explained the observed changes and collected practical suggestions for course refinement. We selected this mixed-methods approach for three reasons: (1) to quantify intervention effects; (2) to clarify pedagogical mechanisms and implementation factors that surveys could not capture; (3) to generate practical feedback for iterative curriculum improvement. This study was reported in accordance with the Good Reporting of A Mixed-Methods Study (GRAMMS) standard (see Supplementary Material S1).

### 2.2. Participants

All full-time undergraduate students who took the course on nursing informatics literacy were included in the study. The exclusion criteria were (a) incomplete survey responses and (b) concurrent enrollment in other nursing informatics-related courses. For the qualitative component, the inclusion criteria were that students had completed the course and consented to be interviewed.

### 2.3. Sample Size Calculation

The required sample size was estimated using GPower (v3.1, Heinrich Heine Universität Düsseldorf, Düsseldorf, Germany) for paired-samples *t*-tests (two-tailed, α = 0.05, power = 0.80). A medium effect size (d = 0.50) was selected based on previous nursing education intervention studies reporting comparable outcomes [30]. The calculation indicated a minimum required sample size of 34 participants. After accounting for a 20% dropout rate, the adjusted minimum required sample size was approximately 43. Ultimately, 120 participants were enrolled. The number of interviewees was determined based on data saturation, defined as the point at which no new themes or insights emerged.

### 2.4. Nursing Informatics Literacy Course Based on the ADDIE Model

We introduced the ADDIE model at the study initiation stage. This ensured that the model guided the entire research process, rather than being introduced mid-course. We applied the ADDIE model to the needs assessment four weeks before the course started. The model then guided all study phases: design, development, implementation, and evaluation. It covered the entire study period and was fully integrated into the whole learning process of undergraduate nursing students. Based on the ADDIE model, the following steps were taken.

#### 2.4.1. Stage 1: Analysis

The teaching team held a meeting to assess training conditions, limitations, and learner needs, and to inform the course design. We identified trends in nursing students’ informatics training through a literature review and policy analysis. Then, a questionnaire survey was conducted to assess students’ evaluations and specific needs in the existing training. A pre-course nursing informatics training needs assessment questionnaire was developed and administered to identify students’ prior experiences, perceived importance, preferred learning styles, and expectations related to informatics education. The questionnaire was constructed based on insights from the preceding literature and policy review and refined through expert discussion within the teaching team. It comprised four sections: (1) background and prior informatics exposure, (2) perceived importance and self-assessed readiness, (3) preferred learning styles and content needs, and (4) perceived barriers and open suggestions. A total of 18 items were included, combining multiple-choice, Likert-type, and open-ended formats. The questionnaire was administered online platform through https://www.wjx.cn/. The website was accessed on 1 March 2025.

The results indicated that the current course emphasized basic computer skills, literature search, and general informatics literacy, but failed to address advanced informatics competency needs. Students preferred hands-on practice, case analysis, and field visits to real-world settings, such as a hospital clinical informatics environment. Key obstacles included a gap between theory and practice and a focus on outcomes over processes. These findings informed the design of a systematic, evidence-based training program.

#### 2.4.2. Stage 2: Design

Guided by needs assessment results, the design phase used a project-based learning framework. We developed a progressive curriculum covering theory, technical skills, and clinical application. Learning objectives targeted mastery of system requirements analysis, process design, and prototype development, plus the integration of informatics into clinical practice and the role of nurses as system designers and optimizers. The curriculum comprised four progressive modules. The first three classes focused on theoretical foundations. They systematically explained the basics of nursing informatics, the methodology of nursing informatics system requirements analysis, and the norms of flowchart design. The fourth through seventh classes strengthened technical practice by covering core skills, such as common interface architecture and function design, in nursing informatics systems. The eighth class conducted project reports to assess students’ competence. The ninth class connected classroom knowledge with clinical scenarios through on-site visits to hospital clinical informatics environments. This formed a closed loop of ‘theory–technology–practice’. Teaching strategies combined case-based learning and project-based learning, embedding real clinical cases in each module (Table 1).

Nursing students are required to complete the entire process, from demand research to prototype design, in groups, thereby achieving capability construction as they solve real problems. In designing the assessment system, an assessment matrix covering knowledge, skills, and attitudes was developed. The system combined quantitative measures (quizzes; self-assessment scales) and qualitative evaluations (project outcomes; course feedback) to comprehensively capture competency gains.

#### 2.4.3. Stage 3: Development

The development phase prioritized clinical applicability by aligning resources, faculty, and practical settings. Firstly, weekly sessions were delivered in multimedia classrooms, featuring lectures, video demonstrations, and case studies, supported by appropriate hardware. Additionally, teaching resources were integrated, such as preparing cases that match the course content and creating video demonstrations of flowchart symbols and draw.io (a web-based chart-drawing tool developed by JGraph) application tutorials to assist students in understanding and practicing. At the same time, the teaching team compiled a standardized report template for students’ nursing informatics system project books, based on the teaching content and objectives. It was used to help students organize details such as the users, requirements analysis, and prototypes of their group’s programs.

#### 2.4.4. Stage 4: Implementation

The instructional team comprised four faculty members, including two consultants, and two teaching assistants. All faculty had over 10 years of nursing informatics experience. They had extensive teaching expertise and a track record of clinical informatics system projects. Both consultants held PhDs in health informatics, had over 20 years of teaching experience, and contributed to the course design. One assistant specialized in nursing informatics; the other held a master’s degree in informatics research. They managed classroom queries, organized materials, assigned tasks, and collected data.

In the first class, the faculty outlined the course objectives and explained the group formation process. Students formed 12 groups of 8–13 members each. The course met weekly for 90 min in a multimedia classroom. Each class comprised 30 min of instruction, 45 min of practice, and 15 min of presentation with feedback. After the theory sessions, each group drafted a design proposal using the provided template, outlining the project’s vision and goals. In the final session, students visited hospital informatics units to observe system-design applications in practice. Teaching methods included lectures, video demonstrations, and case-based learning.

#### 2.4.5. Stage 5: Evaluation

(1) Outcome measures

Primary outcomes

The Self-assessment of Nursing Informatics Competencies Scale: This scale was developed by Staggers et al. [31] and localized and verified by Yu et al. [32]. There are 5 dimensions, including basic computer knowledge and skills, the ability to apply computer skills, informatics role, wireless equipment skills, and nursing informatics attitude, with a total of 28 items. A 5-point Likert scale was used, with higher scores indicating better nursing informatics competencies. The Cronbach’s α values of the Chinese version of the scale were 0.931, the retest reliability was 0.883, and the content validity was 0.950. The Chinese version of the scale had good reliability and validity.

Secondary outcomes

The Informatics Literacy Self-Efficacy Scale: Developed by Kurbanoglu et al. [33], the Chinese version was revised and validated by Huang & Pu [23]. The Chinese version of the scale consists of 21 questions across three dimensions: primary informatics literacy, intermediate informatics literacy, and advanced informatics literacy. The scale was scored on a 7-point Likert scale ranging from 1 (strongly disagree) to 7 (strongly agree). Higher scores reflected greater self-efficacy. The Cronbach’s α coefficient of the scale was 0.942, and the convergent validity and discriminant validity of the scale were good.

The College Students’ Innovative Behavior Measurement Scale: This scale was developed by Jiang et al. [34]. The scale is a four-dimensional concept, including thinking mode, academic exploration, life practice, and academic study. The scale was scored on a 5-point Likert scale, and scores greater than 3 indicated good innovative behavior. The scale had good reliability, with Cronbach’s α coefficients of 0.98 for the overall scale and each dimension.

Course Learning Evaluation Form: This scale was developed by Kim [16] and includes four aspects, namely course content, knowledge, attitude, and project-management skills, with 21 items, and was scored on a 5-point Likert scale. Higher scores denoted greater satisfaction and confidence. The Cronbach’s α coefficient of the scale was 0.99.

Scoring table for outputs and reports from the nursing informatics system project: This scoring table was developed by our research team based on the teaching objectives and content, specifically for evaluating project reports. Student group informatics system projects were handed in one day before they were presented. There were six projects, including project purpose (10 points), user story (10 points), workflow diagram (10 points), functional diagram (30 points), system prototype (30 points), and reporting performance (10 points), with a total of 15 evaluation items. Teachers and two experienced informatics nurses evaluated the accuracy, logic, completeness, rationality, standardization, and esthetics of the projects according to the scoring table. Total scores were converted into letter grades as follows: A (90–100), B (75–89), C (60–74), and D (<60). Content validity of the scoring tool was assessed in this study using a consensus-based content validity index (CVI) method. Five head nurses with more than six years of experience in nursing informatics served as experts to evaluate the scale-level content validity index (S-CVI). The resulting S-CVI was 93.3%.

(2) Data collection

Quantitative data. A trained research assistant managed quantitative data collection. The study’s purpose and participant rights were explained before testing. The pre-test questionnaire was completed three days before the first class, and the post-test questionnaire was completed immediately after the last class. The questionnaire was administered online via https://www.wjx.cn/, with an electronic link to the test and instructions on the first page. The scoring sheets for the project presentations were scored by the judges during the student presentations.

Qualitative data. The interviews were conducted by a researcher trained in qualitative interviewing who was not involved in course grading. At the end of the course, a semi-structured interview was conducted to assess participants’ evaluations of the nursing informatics literacy course using the ADDIE model. When selecting respondents, we considered student gender, nursing informatics competencies, and course scores, and then conducted purposive sampling to ensure a diversity of perspectives. Students were no longer recruited for interviews once no new themes or information emerged (data saturation). To fully understand the teaching effect and students’ real experience of the nursing informatics literacy course based on the ADDIE model, the research team first conducted preliminary interviews with two students and determined the final interview outline: (1) the most beneficial course content; (2) The impact of varied teaching methods and key challenges encountered; (3) perceived improvements in informatics capabilities and their implications for future career development; and (4) suggestions for redesigning course content, format, or assessment. Each interview lasted 20 to 30 min. All interviews were recorded with the participant’s written informed consent. The interviews were transcribed verbatim by the first author within 24 h. Reflexive notes were recorded after each interview, and regular team discussions were held to reduce potential interviewer bias.

### 2.5. Analysis

Quantitative data were analyzed using IBM SPSS Statistics (v26.0, IBM Corp., Armonk, NY, USA). All statistical tests were performed at a two-tailed alpha level of 0.05. The data underwent normality testing before being analyzed. Descriptive statistics, including mean and standard deviation, and analytical statistics, including paired *t*-test, were used. One participant with incomplete baseline data was excluded, and complete-case analysis was applied.

We analyzed qualitative data from semi-structured interviews using thematic analysis [35]. This method includes six steps: data familiarization, coding, theme generation, theme review, theme definition and naming, and report writing. Coding and thematic development were conducted manually using printed transcripts and electronic coding matrices (Microsoft Word (v2021, Microsoft Corp., Redmond, WA, USA) and Microsoft Excel (v2021, Microsoft Corp., Redmond, WA, USA)); no qualitative analysis software was used. Analysis was performed by the first author and another research team member: the first author conducted initial coding for all transcripts, while the second researcher independently coded a randomly selected 25% of the transcripts. To ensure the credibility of the results, we conducted peer debriefings and member checks and held regular meetings to discuss the data analysis process and any discrepancies in opinions. Discrepancies in coding or interpretation were resolved through iterative team discussions until consensus was reached; unresolved disagreements were adjudicated by a senior team member.

To comprehensively analyze the quantitative and qualitative findings, the results were integrated through joint presentations using mixed research methods [36]. We combined and visually displayed the changes in participants’ outcome indicators in the quantitative study and the themes, sub-themes, and related results extracted in the qualitative study in order to compare the findings of different methods and analyze them comprehensively, as well as to enable a more in-depth and comprehensive evaluation of the application and improvement direction of undergraduate nursing information literacy courses.

### 2.6. Ethical Considerations

Ethical approval for this study was granted by the Xiamen Cardiovascular Hospital of Xiamen Institutional Review Board (IRB) under approval number IRB (9) on 28 March 2023, and conducted with the consent of the participating university. In the first week of the course, a researcher explained the study and recruited participants during class. Students were informed of the study’s purpose and methods, and their rights to voluntary, anonymous participation, including the right to withdraw without penalty. All participants provided written informed consent.

## 3. Results

### 3.1. Participant Flow

A total of 120 participants were screened for eligibility, with a 100% attendance rate. One of them did not complete the pre-test and was therefore excluded from the study. Ultimately, 119 participants were included in the final analysis. The participants’ characteristics are reported in Table 2.

### 3.2. Quantitative Results

#### 3.2.1. Pre–Post Outcome Measures

The pre–post outcome measures are reported in Table 3. The total score of nursing informatics competencies before and after the course intervention was statistically significant (*t* = 10.189, *p* < 0.001, Cohen’s d = 0.934, large effect). In addition, before and after the intervention, the total scores for informatics literacy self-efficacy and the ability of students to exhibit innovative behavior were statistically significant (*t* = 9.367, *p* < 0.001, Cohen’s d = 0.859, large effect; *t* = 7.451, *p* < 0.001, Cohen’s d = 0.683, medium effect).

#### 3.2.2. Student Satisfaction with the Project-Based Course

The total score of students’ satisfaction with course learning was (89.03 ± 14.62) (Table 4).

#### 3.2.3. Project Outcomes

All 12 groups completed their projects and delivered oral presentations. Report grades were calculated as the mean of the judges’ scores. Scores ranged from 73 to 91. Of the 12 projects, two received a C, nine received a B, and one received an A. See Supplementary Material S2 for the summary of all 12 projects.

### 3.3. Qualitative Results

Post-intervention interviews yielded three themes and nine sub-themes: (a) cognitive reconstruction and improvement in the ability to apply nursing informatics competencies, (b) dilemmas and breakthroughs in the integration of technology and knowledge in nursing informatics design, and (c) areas for improving course content, design, and evaluation.

#### 3.3.1. Theme One: Cognitive Reconstruction and Improvement in the Ability to Apply Nursing Informatics

This theme captured participants’ cognitive restructuring of nursing informatics competencies during the course. It also reflected their improved ability to use technical tools and manage clinical data. It contained four sub-themes:

##### Informatics Tools Empowered Nursing Practice

Participants reported mastering informatics tools and process-design methods, which they applied to nursing informatics management. These skills enhanced information-management efficiency and supported clinical data processing and system design.

*“I learned to create flowcharts and wireframes with draw.io, enabling systematic nursing-information mapping and procedural app-design exploration.”*

(Participant 1)

*“I learned to use programming tools to process nursing data, such as simple data cleaning and analysis using Python.”*

(Participant 2)

##### Advanced Information Data Management Literacy

Participants recognized that informatics competency extended beyond data collection to include secure storage, analysis, application, and ethical considerations.

*“The course taught me that core informatics functions—orderly storage and rapid retrieval of patient data—support more accurate clinical decisions.”*

(Participant 2)

*“I mastered advanced database queries for efficient retrieval and organization of nursing data.”*

(Participant 3)

*“Implementing permission levels in our app—for example, different data-view scopes for head nurses versus interns—enhanced my privacy-protection awareness.”*

(Participant 4)

##### New Trend in Nursing Informatics Career

Participants were aware of the transformation of the nursing profession to informatics and intelligence, and learned about the emerging career direction of “informatics nurses”. They hoped to participate in optimizing hospital informatics systems and developing nursing apps in the future, becoming a “bridge talent” with both clinical and informatics technology capabilities.

*“The course broadened my view that the nursing profession is not simply clinical nursing. In the future, I can participate in the optimization of hospital informatics systems and develop small applications suitable for clinical practice.”*

(Participant 5)

*“The course let me know the career direction of informatics nurses. I want to combine nursing clinical and informatics technology to become a ‘bridge’ talent who can optimize the nursing process.”*

(Participant 6)

##### User-Centered Design Thinking

Participants said that through case teaching and project practice, they learned to design nursing informatics systems from a user perspective and to focus on user needs analysis and experience optimization.

*“When designing the flow chart, stand in the user’s perspective and understand the logic of the informatics application.”*

(Participant 7, Participant 8)

*“The user experience should be taken into account when designing the APP, such as the size of the button should be suitable for finger clicking, otherwise nurses and patients will be reluctant to use it.”*

(Participant 9)

*“Interviews can be added before the design to understand the needs of patients and improve the practicality of the design.”*

(Participant 1)

#### 3.3.2. Theme Two: Dilemmas and Breakthroughs in the Integration of Technology and Knowledge in Nursing Informatics Design

This theme is mainly related to the challenge of integrating technology and knowledge faced by participants in the learning process of the course, including two sub-themes:

##### Technical Implementation and Tool Application Barriers

Some participants reported that during the course, they encountered technical operational problems, primarily in three areas: flowchart/wireframe design, programming implementation, and software operation. Nursing participants generally lack an informatics technology base, and there is a clear fault line in translating care needs into technology solutions.

*“Programming function code writing difficulties, need to consult a lot of information, consult teachers.”*

(Participant 10)

*“The biggest challenge is flow charting.”*

(Participant 11)

*“Difficulty with drawing tools.”*

(Participant 12)

##### Difficulties in Multidisciplinary Knowledge Integration

Some participants said that nursing informatics design needed to integrate multidisciplinary knowledge, such as nursing, informatics technology, and user experience design, but there were significant difficulties in integrating and applying interdisciplinary knowledge.

*“The design of nursing informatics projects should integrate the perspectives of nursing, informatics technology, and user experience design. When designing APP interfaces, IT is necessary to balance medical data security norms and patient operation convenience, which involves nursing, IT, and design knowledge.”*

(Participant 4)

*“Case analysis involves multidisciplinary knowledge, and it is difficult to comprehensively analyze with nursing knowledge alone.”*

(Participant 13)

#### 3.3.3. Theme Three: Optimization Direction of Course Content, Design, and Evaluation

This theme was mainly related to the optimization direction of the content, design, and evaluation of the nursing informatics literacy course, and contained three sub-themes:

##### Optimization of Content Combining Theory and Practice

Participants emphasized the importance of “hands-on operation” and “complete project experience”. They suggested adding practical links, updating the course content to supplement emerging technologies and interdisciplinary content, and strengthening the connection between the course and clinical scenarios.

*“More hands-on practice and hospital visits would clarify practical system workflows.”*

(Participant 14, Participant 15)

*“I hoped to increase the application cases of AI in nursing, which is very helpful for clinical work. In addition, the application and learning of blockchain technology in nursing data security and privacy protection were increased, and the awareness of information protection was strengthened.”*

(Participant 2, Participant 16)

*“Integrating psychological principles would improve user-need understanding and product acceptability.”*

(Participant 17)

##### Ability Symbiosis and Challenge Response in Teamwork

Participants valued project-based group learning for enhancing teamwork and communication, but encountered disparities among members, coordination challenges, and uneven task distribution difficulties.

*“Differences in knowledge and technical skill led to disagreements over app design and task assignment, requiring repeated discussion. Scheduling conflicts further stalled project progress.”*

(Participant 5)

*“Through the design of the APP through group cooperation, I have learned how to apply theoretical knowledge to practical projects, and deepened my understanding of application program design from requirements analysis, functional design, to interface layout and other links. Case teaching allows me to see the application of nursing informatics systems in different scenarios and broaden my horizon.”*

(Participant 6)

##### Diversified Assessment and Blended Teaching

Participants suggested that diversified assessment methods should be adopted, and that individual practice assessment and group mutual evaluation should be added to address unequal abilities and contributions among members during group collaboration. Moreover, they recommended diverse teaching modalities to boost engagement and learning efficacy.

*“Timed tasks (e.g., electronic health records data extraction and analysis) could assess practical competence.”*

(Participant 4)

*“A hybrid model with online tutorials, offline workshops, and expert guidance would expedite skill acquisition.”*

(Participant 5)

*“The mechanism of mutual evaluation within the group was introduced to improve the ability of team collaboration and reflection.”*

(Participant 4)

### 3.4. Integration of Quantitative and Qualitative Results

Table 5 shows the results of the analysis of the quantitative and qualitative data. The quantitative and qualitative findings were consistent and complementary. The integration of quantitative and qualitative findings revealed three key patterns. First, convergence was observed, as both quantitative improvements in competencies and qualitative reports of enhanced skills consistently preliminarily indicated positive learning outcomes. Second, complementarity was identified, with qualitative findings providing detailed explanations for how students developed confidence and applied informatics knowledge in practice. Third, expansion was evident, as qualitative data highlighted challenges such as technical barriers and interdisciplinary integration that were not captured by the quantitative measures.

## 4. Discussion

### 4.1. Summary of Key Findings

To our knowledge, this is the first mixed-methods study to develop and pilot an ADDIE-based nursing informatics literacy course for undergraduate nursing students. Our quantitative findings suggested that participation in the course may have been associated with improvements in nursing informatics competencies, informatics literacy self-efficacy, and innovative behavior. These findings should be interpreted as preliminary signals rather than definitive evidence of effectiveness. The qualitative results indicated that the program was perceived as acceptable and feasible by participants, who reported enhanced understanding of informatics applications and identified areas for course refinement.

### 4.2. Comparison with Previous Research

The quantitative and qualitative results both showed preliminary improvements in informatics competency, self-efficacy, and innovative behavior. These changes were not just score increases—they reflected meaningful progress in students’ learning and skill development. These findings were consistent with recent international evidence indicating that structured, technology-enhanced, and experiential nursing informatics education can improve students’ informatics competency and readiness for digital health practice [14,25,30,37]. Several factors may have accounted for these observed improvements. First, the ADDIE-based progressive curriculum was targeted and systematic. We designed it around participant characteristics and training objectives. Instructional plans were grounded in the guided implementation of the principle of “combining learning with practice”. Meanwhile, the five ADDIE phases were tightly integrated, with each phase’s output informing the next, reinforcing curriculum coherence. Consequently, the curriculum addressed prior gaps in clinical integration and process-oriented learning [29]. Qualitative data showed that students’ cognitive reconstruction of nursing informatics competence and their need for knowledge regarding practice transformation further supported the effectiveness of the course design. This aligns with Luo et al.’s [38] finding that a phased ADDIE design enhances theoretical and practical competencies. Echoing the TIGER competency framework, this requirements-driven design translated abstract competencies into actionable modules [8]. Given the observed benefits of early exposure to the ADDIE model in nursing students’ professional development, nursing programs were encouraged to integrate this ADDIE-based course into the early undergraduate curriculum. A progressive, practice-oriented delivery format was also recommended to sustain continuous improvement in nursing informatics competencies.

Second, clinical case studies and hospital visits created authentic learning environments. Participants observed nurse-led informatics system design and applied these competencies in practice, consistent with Bandura’s distinction between direct and indirect experience. These field visits deepened their understanding of medical units’ information needs and highlighted emerging digital health paradigms driven by nursing priorities [30]. Third, the project-based system design aligned with constructivist learning theory. This approach promoted deep learning through real-world problem solving [39]. Twelve group projects covered the entire process from demand investigation to prototype design and promoted the integration of nursing informatics competencies through a “learning by doing” approach. Qualitative data confirmed that this approach led to cognitive reconstruction and improved nursing informatics competence, echoing Sockolow’s and Bowles’s [15] informatics project-management strategy. Fourth, requiring informatics system design for clinical scenarios fostered demand-driven innovation. Professional knowledge has been shown to underpin student creativity. In this study, participants acquired nursing informatics expertise, enabling them to complete innovative projects [24]. Previous studies had found a positive correlation among informatics literacy, informatics competence, and innovative behavior [24,25]. Innovative behavior is the value externalization of the former two in clinical scenarios. The improvement in innovative behavior may have been related to the integration of knowledge and practice in project-based tasks, which encourages students to apply informatics skills to real-world problems and supports creative problem-solving [24]. Bandura’s self-efficacy theory also pointed out that self-efficacy affected behavioral engagement through “choice effect” and “effort effect” [40]. The concurrent improvements across the three outcomes may be consistent with this theoretical perspective; however, the underlying relationships were not directly tested in this study. In addition, although self-efficacy, competencies, and innovative behavior were assessed simultaneously, their interrelationships were not formally examined. Future studies can use mediation analysis, structural equation modeling, or longitudinal designs to test these pathways.

Last but not least, the preliminary effect of this course on students was due to the support of a strong teaching team. Previous studies have shown that a strong teaching staff is key to improving students’ nursing informatics competencies [10,18]. Our team comprised faculty, professional consultants, and teaching assistants with extensive backgrounds and practical experience in nursing informatics. Since 2015, our hospital has implemented activities for training and development in nursing informatics competency [41], culminating in the professional-practice-model-based nursing informatics competency organization (NICO-PPM) model [42]. Using the NICO-PPM model, we have developed specific practices and achievements in terms of training nursing informatics talent, conducting and publishing research, obtaining patents, and attending international conferences [43]. However, many countries—including Sweden, South Korea, and China—face shortages of nursing informatics faculty [10]. Therefore, more attention should be paid to the construction of nursing informatics teachers in the future.

Student satisfaction was high across course content, knowledge acquisition, attitude change, and project management, indicating good acceptability of the intervention. This positive feedback may be related to the course’s learner-centered, practice-oriented design. The qualitative findings further suggested that students valued opportunities to apply knowledge in realistic tasks. Similar associations between active learning design and learner satisfaction have been reported in recent nursing education studies [14].

Notably, the qualitative study showed that students had developed user-centered design thinking. This aligns with the nursing field’s “patient-centered” technology paradigm and underscores the course’s preliminary effectiveness in fostering design thinking, which is often neglected as a key element of nursing informatics education [44]. Additionally, participants reported a shift in their perception of professional identity. Their understanding of the role of nurses shifted from “passive technology user” to “active system designer”, consistent with the international trend of reshaping the role of nurses in digital health [45,46]. Participants also recognized the importance of information security and ethical standards, consistent with evolving industry benchmarks in data-driven healthcare [47].

However, participants identified areas for course refinement. First, they faced technical operation barriers and challenges when integrating multidisciplinary knowledge. It was suggested to increase hierarchical technical training and interdisciplinary faculty collaboration, and to include AI applications, blockchain technology, and other emerging and interdisciplinary content. This aligns with the emphasis of the WHO’s Global Digital Health Strategy 2020–2025 on digital skills education [48] and reflects trends toward intelligent nursing informatics, which previous undergraduate courses had underemphasized [49,50]. Second, participants advocated for individual assessments and peer evaluations to address disparities in group collaboration and contribution. This approach would enable more accurate individual assessment and promote peer accountability, enhancing overall learning outcomes. Third, participants recommended diverse teaching modalities to boost engagement and learning efficacy [51]. Similarly, a study examining the impact of integrating clinical simulation scenarios and informatics technology into nursing skills teaching indicated that using multiple innovative training formats, including flipped classrooms, clinical simulation scenarios, and blended learning, could improve nursing students’ learning outcomes [52].

### 4.3. Strengths and Limitations

This study provided a relatively comprehensive perspective on integrated “Nursing + X” courses through a mixed-methods design. Through systematic teaching design, real-world practice, and multidimensional evaluation, the course may help support the development of nursing informatics talent with both clinical insight and technical innovation. However, this study had several methodological limitations. The study used a single-arm pre-test–post-test design with no control group. This limits our ability to draw causal conclusions about the intervention’s effects. The improvements observed may have been partially influenced by maturation, testing effects, or other contextual factors. Therefore, the findings should be interpreted with caution. Second, generalizability should also be considered cautiously. The study was conducted in one university within a specific educational context, with a locally developed curriculum and a relatively homogeneous student population. The findings may therefore not directly transfer to institutions with different resources, curricula, or cultural learning environments. Third, outcomes were assessed immediately after the intervention. The absence of follow-up assessment limits conclusions regarding long-term impact. It remains unclear whether short-term improvements would be sustained over time or translated into future clinical informatics practice after graduation. Finally, as participants were first-year nursing students with limited clinical exposure, the findings may not be directly applicable to students at later stages of training. These limitations should be considered when interpreting the results. Future studies should employ multicenter randomized controlled trials to compare ADDIE-based and traditional courses and to longitudinally track graduates’ informatics competencies and innovative behaviors.

## 5. Conclusions

This study found that an ADDIE-based nursing informatics literacy course may have been associated with improvements in nursing informatics competencies, informatics literacy self-efficacy, and innovative behavior among undergraduate nursing students. These findings provide preliminary evidence supporting the feasibility and potential value of structured informatics education, but confirmation in controlled studies is required.

## Figures and Tables

**Table 1:** Content of nursing informatics literacy course.

Class	Theme	Objective	Theoretical Content	Practice Content	Teaching Strategy
1	Overview of Related Concepts	To understand related concepts of nursing informatics	(1) Nursing informatics and nursing informatics competencies (2) What is project management	/	To understand related concepts of nursing informatics
2	Needs Analysis	To master the methods and techniques of needs collection, sorting, and analysis	(1) User self-built concept (2) Needs analysis methods, such as context interview method, user story method, etc. (3) Skillfully using the user story comparison method to sort out functions (4) Minimum viable product (5) Kano model	(1) Develop an initial project concept, including user roles, objectives, and scope (2) Write the user story object and data collection framework of the group system	Lecturing, case-based learning
3	Needs Analysis	To master informatics workflow, re-engineering, and functional flowchart drawing	(1) Common symbols and drawing of flowcharts (2) Common flowchart types (3) Functional flowchart and interface flowchart drawing	(1) Draw a functional flowchart (computer or hand-drawn) for a certain function generated from the user story	Lecturing, case-based learning
4	Application Design	To master common interface architecture and functional design	(1) Functional architecture disassembly (2) Brief introduction to system architecture	Independently select an application program for disassembly and architecture interpretation	Lecturing, direct demonstration, case-based learning
5	Application Design	To master interface design specifications	(1) Design specifications for user interface (UI) and user experience (UX) (2) Prototype concept (3) Interface design key points, such as page types, interface colors, and color-matching tools, and common components	Draw part of the interface of the group’s application program (computer or hand-drawn)	Lecturing, direct demonstration, case-based learning
6 and 7	Application Design	To draw prototype interface diagrams with the aid of tools	Auxiliary tools for drawing interface prototypes, such as draw.io (v24.7.4, JGraph Ltd., London, UK), and operation skills	Use draw.io to draw program prototypes (computer)	Lecturing, direct demonstration, case-based learning
8	Project Report	To evaluate students’ mastery	/	Organize the group’s project report according to the report template	Project report
9	Nursing Informatization	To emphasize the development of meaningful clinical informatics systems based on nursing needs	(1) Introduction to hospital nursing informatics systems: mobile nursing informatics systems, PDAs, follow-up systems, intelligent medicine cabinets, visual electronic whiteboards, etc. (2) Survey feedback	Hospital visit	

**Table 2:** Characteristics of participants (*n* = 119).

Characteristics		n (%)
Gender	Male	29 (24.4%)
	Female	90 (75.6%)
Age (years), mean (SD)	Mean (SD)	18.84 ± 0.736
	Range	17–21
Have you taken any computer-related courses?	No	15 (12.6%)
	Yes	104 (87.4%)
National computer rank examination certificate	None	104 (87.4%)
	Grade 1	15 (12.6%)
Ever heard of nursing informatics?	No	63 (52.9%)
	Yes	56 (47.1%)

**Table 3:** Pre–post outcome measures (n = 119, score).

Variables	Baseline (Mean ± SD)	Post-Intervention (Mean ± SD)	*t*	*p*	Cohen’s d
Nursing informatics competencies	62.97 ± 17.88	80.08 ± 20.15	10.189	<0.001	0.934
Informatics literacy self-efficacy	94.51 ± 17.77	111.25 ± 16.99	9.367	<0.001	0.859
Innovative behavior	3.31 ± 0.58	3.74 ± 0.56	7.451	<0.001	0.683

**Table 4:** Student satisfaction with project courses.

Dimension	Score
Total scores	89.03 ± 14.62
Course content	21.42 ± 3.08
Knowledge	21.12 ± 4.54
Attitude	25.26 ± 4.98
Skill in project management	21.23 ± 5.03

**Table 5:** Joint display of quantitative and qualitative results.

Outcome	Quantitative	Qualitative	Integration
Nursing informatics competencies	Significant improvement (*p* < 0.001, *d* = 0.934).	Improved tool use, workflow thinking, and professional identity	Complementarity and Expansion: The intervention drove meaningful competency gains while exposing key implementation bottlenecks, revealing a gap between skill improvement and real-world application readiness.
Informatics literacy self-efficacy	Significant improvement (*p* < 0.001, *d* = 0.859).	Greater confidence in information use and problem-solving	Complementarity: Qualitative findings contextualize the observed quantitative improvement, suggesting hands-on practice may have contributed to enhanced informatics literacy self-efficacy.
Innovative behavior	Significant improvement (*p* < 0.001, *d* = 0.683).	Creative prototype design and solution generation	Complementarity: Quantitative improvements in innovative behavior are supported by qualitative findings, which show that these gains were expressed through creative prototype design and solution generation in demand-driven projects.
Student satisfaction	Overall satisfaction score: 89.03 ± 14.62.	Positive feedback on project-based learning; suggestions for emerging technologies and diversified assessment	Complementarity and Expansion: The ADDIE-based nursing informatics course received robust student acceptance, with targeted refinements identified for technological integration and evaluation frameworks.
Project outcomes	12 projects (1 A, 9 B, 2 C).	Projects showcased knowledge application, but faced technical implementation and multidisciplinary integration challenges	Expansion: Competent knowledge application in projects was paired with implementation challenges, highlighting the need for skill reinforcement and course refinement.

## Data Availability

The data that support the findings of this study are available on request from the corresponding author, Y.C. The data are not publicly available due to privacy and ethical restrictions imposed by the institutional review board.

## Supplementary Materials

The following supporting information can be downloaded at: https://www.mdpi.com/article/10.3390/nursrep16050151/s1, Supplementary Material S1: GRAMMS (Good Reporting of A Mixed-Methods Study) checklist. Supplementary Material S2: Project Outcomes. Ref. [53] is cited in the supplementary material file.

## Public Involvement Statement

There was no public involvement in any aspect of this research.

## Guidelines and Standards Statement

This study was reported in accordance with the Good Reporting of A Mixed-Methods Study (GRAMMS) standard.

## Abbreviations

The following abbreviations are used in this manuscript:

ADDIE	Analysis, Design, Development, Implementation, and Evaluation
