# Supplementary material for: Design, Implementation, and Preliminary Evaluation of an Undergraduate Nursing Informatics Literacy Course Based on the ADDIE Model: A Single-Arm Mixed-Methods Study
Source: Nurs Rep. 2026 Apr 28;16(5):151. doi: 10.3390/nursrep16050151 (PMC13209881; doi:10.3390/nursrep16050151)
Supplement: Supplementary file 1 [file nursrep-16-00151-s001.zip › Supplementary Material S1 GRAMMS (Good Reporting of A Mixed Methods Study) checklist..pdf]

Good reporting of a mixed-methods study (GRAMMS) checklist

Guideline	Page information
1. Describe the justification for using a mixed methods approach to the research question	5-6
2. Describe the design in terms of the purpose, priority and sequence of methods	5-6
3. Describe each method in terms of sampling, data collection and analysis	6-15
4. Describe where integration has occurred, how it has occurred and who has participated in it	14-15 , 22 - 23
5. Describe any limitation of one method associated with the present of the other method	27-28
6. Describe any insights gained from mixing or integrating methods	23-28

Reference: O'Cathain A, Murphy E, Nicholl J. The quality of mixed methods studies in health services research. J Health Serv Res Policy. 2008;13: 92-98.
