# Supplementary material for: Design, Implementation, and Preliminary Evaluation of an Undergraduate Nursing Informatics Literacy Course Based on the ADDIE Model: A Single-Arm Mixed-Methods Study
Source: Nurs Rep. 2026 Apr 28;16(5):151. doi: 10.3390/nursrep16050151 (PMC13209881; doi:10.3390/nursrep16050151)
Supplement: Supplementary file 1 [file nursrep-16-00151-s001.zip › Supplementary Material S2 Project Outcomes..pdf]

Project Outcomes

All 12 groups completed the projects and made an oral presentation.

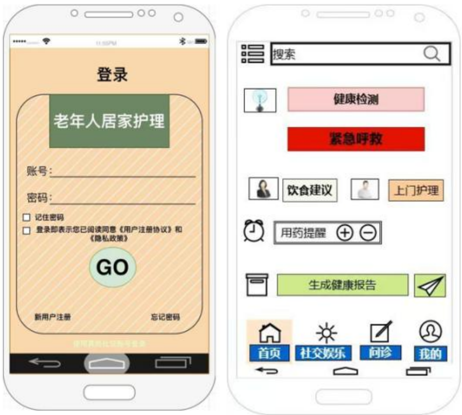

Application interface diagram

1. Home care for the elderly

It aims to meet the needs of the elderly to receive care at home and improve their quality of life by providing professional home care services.

Average score: 82

Grade: B

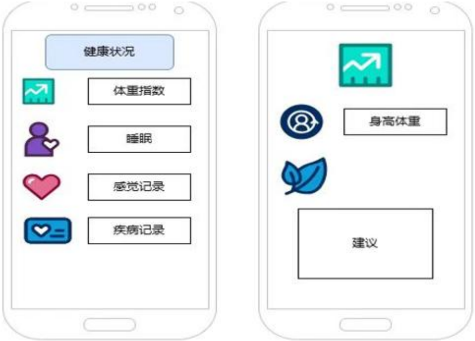

Application interface diagram

2. Mulberry elm is happy to keep

To provide a comprehensive platform for the elderly with health management, daily care, entertainment and leisure functions.

Average score: 77

Grade: B

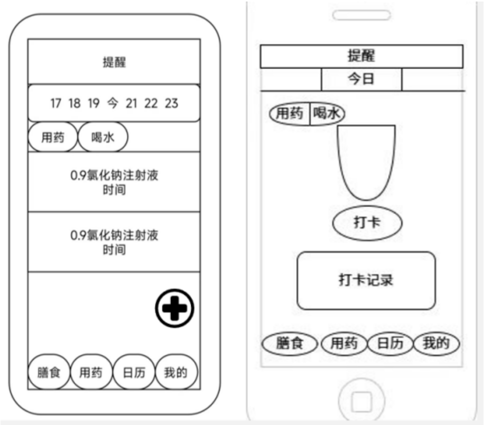

Application interface diagram

3. Department of Food and Medicine

The purpose is to personalize the daily meal plan for users and remind the sick users to take medicine on time and according to the amount.

Average score: 86

Grade: B

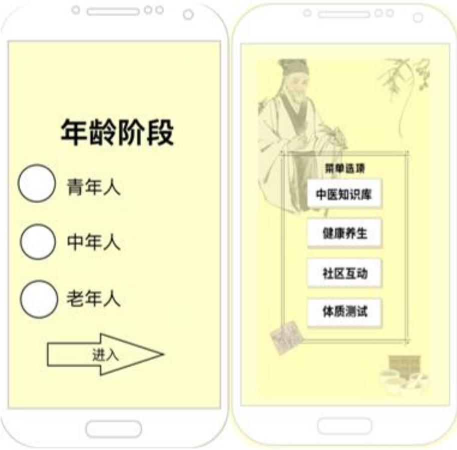

Application interface diagram

4. Bid farewell to "crispy skin" self-health of traditional Chinese medicine

> The purpose is to learn traditional Chinese medicine knowledge for users of different ages and develop healthy living habits.

Average score: 91

Grade: A

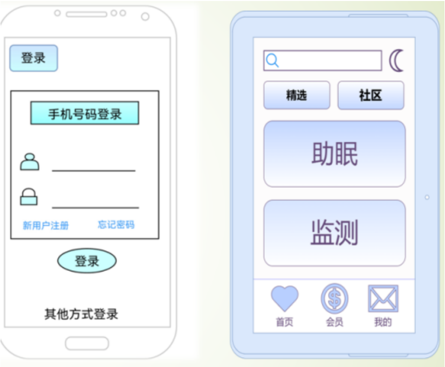

Application interface diagram

5. Sleep quality measurement

> It aims to promote users' sleep and improve sleep quality.

Average score: 76

Grade: B

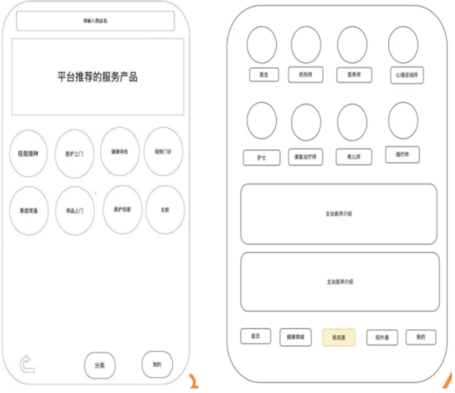

Application interface diagram

6. Palliative care

> It aims to improve the quality of life of patients, promote the mental health of family members, and promote the development of the industry.

Average score: 73

Grade: C

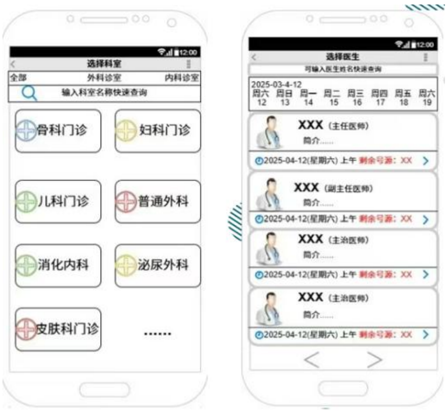

Application interface diagram

7. Smart care

The aim is to detect patient health, reduce nurses' workload and save doctors' time.

Average score: 75

Grade: B

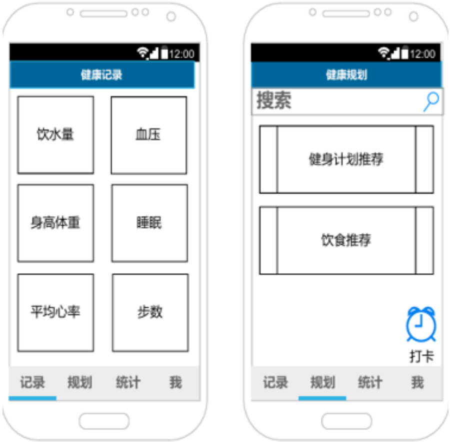

Application interface diagram

8. Daily healing

It aims to help users maintain and improve personal health more scientifically and efficiently through digital tools. It is characterized by a shift from "passive treatment" to "active health management".

Average score: 74

Grade: C

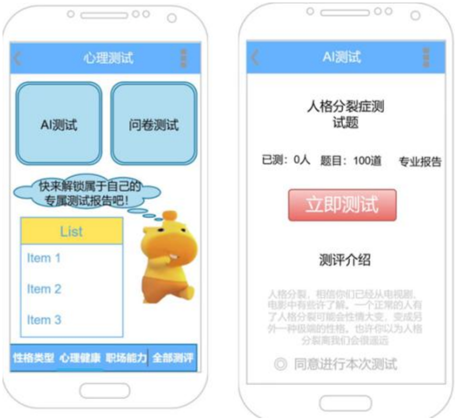

Application interface diagram

9. Healing the heart

It aims to fill gaps in mental health services, remove barriers to help-seeking, and personalize mental health management.

Average score: 80

Grade: B

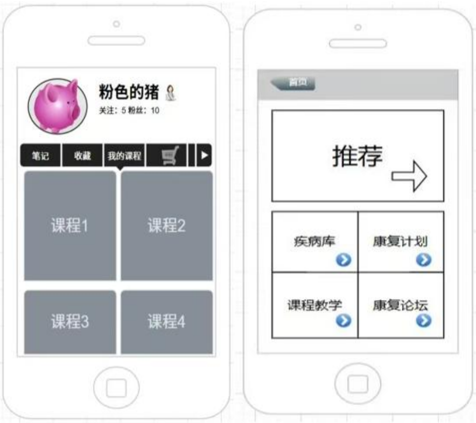

Application interface diagram

10. Benefit of rehabilitation

The purpose is to provide users with rehabilitation knowledge popularization, make rehabilitation plans, and show rehabilitation progress.

Average score: 84

Grade: B

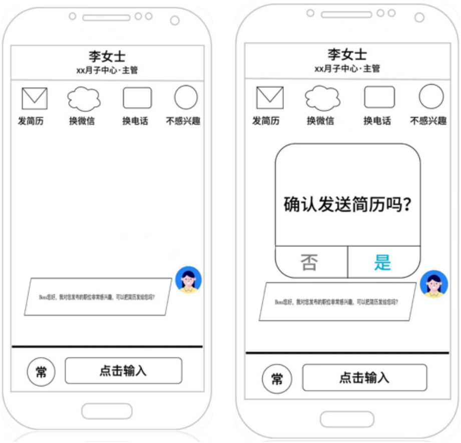

Application interface diagram

11. Job search

It aims to connect job seekers and enterprises to achieve efficient talent matching. And the two-way realization of personal and social values.

Average score: 80

Grade: B

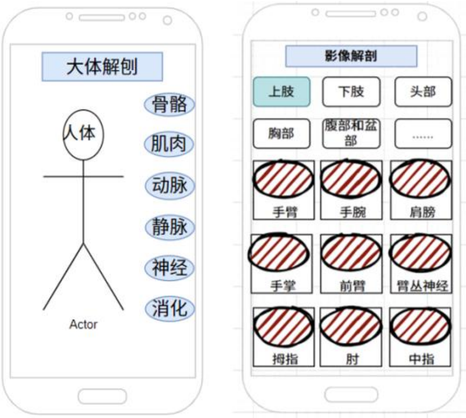

Application interface diagram

12. Virtual human anatomy

To improve medical students' anatomical knowledge, facilitate teachers' teaching, and help the people understand anatomical knowledge.

Average score: 80

Grade: B
